# Kappa free light chains index in multiple sclerosis very long-term prognosis

**DOI:** 10.3389/fimmu.2023.1223514

**Published:** 2023-10-11

**Authors:** Pablo Arroyo-Pereiro, Lydia García-Serrano, Francisco Morandeira, Blanca Urban, Virginia Mas, Mario Framil, Isabel León, Albert Muñoz-Vendrell, Elisabet Matas, Lucía Romero-Pinel, Antonio Martínez-Yélamos, Sergio Martínez-Yélamos, Laura Bau

**Affiliations:** ^1^ Multiple Sclerosis Unit, Department of Neurology, Hospital Universitari de Bellvitge- Institut d’Investigació Biomèdica de Bellvitge (IDIBELL), L’Hospitalet de Llobregat, Spain; ^2^ Department of Immunology, Hospital Universitari de Bellvitge – Institut d’Investigació Biomédica de Bellvitge (IDIBELL), L’Hospitalet de Llobregat, Spain; ^3^ Departament of Clinical Sciences, Facultat de Medicina i Ciències de la Salut, Universitat de Barcelona (UB), Barcelona, Spain

**Keywords:** multiple sclerosis, kappa free light chains, biomarkers, long-term, prognosis, cerebrospinal fluid, disability

## Abstract

**Introduction:**

The role of the kappa-free light chain (kFLC) in the diagnosis of multiple sclerosis (MS) and, to a lesser extent, its role as a medium-term prognostic marker have been extensively studied. This study aimed to explore its potential as a long-term prognostic marker for MS.

**Methods:**

We performed an exploratory retrospective observational study by selecting patients systemically followed up in our MS unit with available cerebrospinal fluid and serum samples at the time of initial evaluation. Two groups were defined: benign MS (bMS), defined as patients with Expanded Disability Status Scale (EDSS) ≤ 3 at 10 years of follow-up, and aggressive MS (aMS), defined as patients with EDSS ≥ 6 at 15 years of follow-up. Clinical variables were collected, and the immunoglobulin G (IgG) index, kFLC index, and oligoclonal bands (OCB) were determined for all patients and compared between the groups.

**Results:**

Twenty bMS and 15 aMS patients were included in this study. Sixty percent (21/35) were female, and the mean age at the time of the first symptom was 31.5 ± 9.45 years, with no statistical differences between groups. Median follow-up time was 19.8 years (Interquartile range, IQR 15.9–24.6). The median EDSS scores at the last follow-up were 1.5 and 7.5 in the bMS and the aMS group, respectively. No statistically significant differences were found in the kFLC index between the two groups (136.6 vs. 140.27, p=0.59). The IgG index was positive in 62.9% of patients (55% bMS vs. 73.3% aMS, p>0.05), and OCB was positive in 88.6% (90% bMS vs. 86.7% aMS, p>0.05). A significant positive correlation was found between IgG and kFLC indices (r_s_ = 0.85, p<0.001).

**Conclusion:**

Given the absence of differences between the two groups with opposite disease courses, it is unlikely that the kFLC index is a reliable and powerful marker of long-term prognosis in MS.

## 1 Introduction

Multiple sclerosis (MS) is a chronic inflammatory immune-mediated neurological disease with a variable course derived from the accumulation of disability as a consequence of inflammatory relapses and progression independent of relapses (1).

Thus, with the individual course of the disease being highly variable, there are currently only a few prognostic markers for practical use (2, 3). A recent approach to treatment using early high-efficacy treatments versus the more traditional approach in which treatment is scaled up progressively to prevent the accumulation of disability in patients with aggressive forms has generated the need for prognostic markers; therefore, stratification can be made to guide treatment decisions (4–6).

MS is classically considered an organ-specific autoimmune disease mediated mainly by T lymphocytes, although the role of B lymphocytes is considered important as evidenced in animal models (7, 8), by pathological studies in MS (9), by the presence of oligoclonal bands (OCB) in the cerebrospinal fluid (CSF) (10) and indirectly by the efficacy of targeted therapies against B cells (11), among other data.

Free light chains (FLC) are a product of the physiological activity of plasma cells, which in their immunoglobulin production process generate an excess of light chains (kappa or lambda) that are not incorporated into their usual tetrameric structure. They can be interpreted as markers of the activity of plasma cells and are detectable in various fluids (12–15). Several metrics have been proposed to consider the permeability of the blood-brain barrier and therefore more accurately reflect the intrathecal fraction of FLC (16), with the FLC index being ultimately the most widely used.

Lately, the usefulness of the kappa FLC (kFLC) index in the diagnosis of MS has been extensively studied, and its equivalence to OCB has been suggested by various studies (17–19), although some studies disagree (19). A few recently published studies using the kFLC index and modern nephelometry or turbidimetry techniques have aimed to establish its potential role as a prognostic marker. However, its role in long-term prognosis has not been explored (20–22).

We hypothesized that the kFLC index could predict long-term MS outcomes. Therefore, this study aimed to assess kFLC in two groups with opposite clinical courses to evaluate its potential role as a long-term prognostic marker in MS.

## 2 Materials and methods

### 2.1 Study design, patients and clinical data collection

In this proof-of-concept study, an exploratory retrospective observational design was used, including patients diagnosed with Relapsing Remitting MS according to the McDonald criteria of 2017 (23) who were systematically followed up in the MS unit of a tertiary hospital with available CSF and serum samples at the time of the initial patient evaluation.

Two groups were defined with distinctive inclusion criteria: benign MS (bMS), defined as patients with Expanded Disability Status Scale (EDSS) ≤ 3 at 10 years of follow-up, and aggressive MS (aMS), defined as patients with EDSS ≥ 6 at 15 years of follow-up.

Patients were excluded if they had renal function impairment, the presence of mono-or polyclonal gammopathies, the presence of other neoplasms, the presence of any systemic or inflammatory disease (including sarcoidosis, lupus, rheumatoid arthritis, Sjögren syndrome, allergy, chronic rhinitis, asthma, idiopathic pulmonary fibrosis, interstitial hypersensitivity pneumonitis, or HIV infection), presence of any other central nervous system diseases, or treatment with corticosteroids in the month prior to lumbar puncture (LP) performance and blood sampling. Patients in whom the samples did not meet the technical criteria (presence of particles, lipemia, or hemolyzed samples) were also excluded. The time from onset of the disease to LP was not consider as an exclusion criteria.

The European Database for Multiple Sclerosis software was used for the prospective collection of clinical data (24). The variables collected were sex, date of birth, date of MS onset, date of LP, date of last relapse prior to LP, date of last dose of corticosteroid prior to LP, date of last follow-up, total number of relapses during follow-up, EDSS at the time of LP, EDSS at 10 years of onset for bMS, EDSS at 15 years of onset of aMS, EDSS at the end of follow-up for all patients, disease-modifying treatment (DMT) received before LP and during follow-up, and progression start date.

The following variables were calculated: age at onset, age at first LP, total follow-up time, time from MS onset to LP, and time from relapse to LP. The DMT variable was analyzed as: “no DMT”; “moderate efficacy DMT” if any of the following was used at any moment of the follow-up and none of the high efficacy DMT: interferon beta 1a, interferon beta 1b, peginterferon beta 1a, glatiramer acetate, teriflunomide, dimethyl fumarate, fingolimod, cladribine; and “high efficacy DMT” if any of the following was used at any moment of the follow-up: natalizumab, alemtuzumab, ocrelizumab, rituximab, mitoxantrone. The diagnostic criteria for secondary progressive multiple sclerosis (SPMS) used to define the progression date are those previously defined in 2016 and widely used (25).

### 2.2 Samples and laboratory analysis

Patient samples were provided by Biobank HUB-ICO-IDIBELL, funded by the Instituto de Salud Carlos III (PT20/00171), and by Xarxa de Bancs de Tumors de Catalunya, sponsored by Pla Director d’Oncologia de Catalunya (XBTC). Samples were collected as part of the usual initial patient evaluation and stored at -80°C until analysis.

The following parameters were determined in both the serum and CSF: albumin, total immunoglobulin G (IgG), kFLC, and OCB.

IgG OCB was detected by isoelectric focusing on a Hydrasys Focusing System (Sebia Hispania, Barcelona, Spain) according to the manufacturer’s instructions. OCB were considered positive if at least two bands in the CSF were present without corresponding bands in the serum. IgG and albumin levels in the CSF and serum were measured using a BN II nephelometer system (Siemens Healthcare, Marburg, Germany). To account for the influence of blood barrier permeability, the IgG index was calculated using the following formula:


$$IgG\;index=\frac{CSF\;IgG\times serum\;Albumin}{serum\;IgG\times CSF\;Albumin}$$

CSF and serum KFLC levels were measured by turbidimetry using the Freelite Mx Free Kappa Optilite kit (Binding Site, Birmingham, UK) on an Optilite Analyzer (Binding Site). The kFLC quotient and index were calculated using the following formulae:


$$KFLC\;quotient=\frac{CSF\;KFLC}{serum\;KFLC}$$

$$KFLC\;index=\frac{CSF\;KFLC\times serum\;Albumin}{serum\;KFLC\times CSF\;Albumin}$$

In those samples with a kFLC value below the detection threshold (<0.33 mg/L according to the manufacturer), a value of 0.17 mg/L was assigned to them.

Values of IgG index > 0.7 were considered as positive.

### 2.3 Study endpoints

The primary endpoint was the difference in kFLC index between patients with bMS and those with aMS. As secondary endpoints, differences in the IgG index and OCB status were assessed between both groups, and the correlation between kFLC and IgG indices was analyzed.

### 2.4 Statistical analysis

Data are described as mean ± standard deviation (SD) or as median and interquartile range (IQR), according to their distribution. Categorical variables are described using frequencies. The distribution of the data was assessed using the Shapiro-Wilk W test. For group comparisons, the chi-square test, Fisher’s exact test, Mann-Whitney U test, and negative binomial regression were applied as appropriate. Spearman’s rank correlation coefficient was used to assess the correlation between the kFLC and IgG indices, the time from MS onset to LP, and the time from previous relapse to LP. To assess the biomarkers accuracy in classifying patients into benign or aggressive forms, receiver operating characteristic (ROC) curves and derived area under the curve (AUC) were calculated. ROC curve standard error calculations and comparisons were performed with DeLong’s method All tests were conducted with 95% confidence intervals (CIs) and a significance level of 5%. Statistical analyses were performed using the Stata 13 software (StataCorp LLC, Texas, USA).

### 2.5 Ethics approval

The study was approved by the Ethics Committee of the Hospital Universitari de Bellvitge (reference PR134/22). Patient information confidentiality was addressed in accordance with the Spanish regulations.

## 3 Results

### 3.1 Groups characteristics

Out of the 2,216 patients included in the EDMUS database, 206 patients met the criteria of aMS group, of which 16 cases had available paired CSF and serum samples from the initial study. 1 had to be excluded due to medical history of chronic allergy. A total of 729 patients met the criteria for bMS. Among these, 20 patients age-matched patients with paired CSF and serum samples were selected. Twenty patients were included as having bMS and 15 as having aMS. Sixty percent were female, and the mean age at MS onset was 31.5 ± 9.45 years old. They were followed up for a median of 19.8 years (IQR 15.9-24.6). No statistical differences were found in age at onset nor in follow-up time between groups. In the latter although deemed no statistically significant, the median follow-up was approximately 4 years longer for the aMS group (17.33 in bMS vs. 21.6 years in aMS; p=0.06). In this regard, despite the fact that the time from MS onset to LP was not statistically different between groups (24.21 in bMS vs. 39.49 months in aMS; p=0.3), age at the time of LP was indeed higher for aMS (31.05 in bMS vs. 39.69 years in aMS; p=0.018). The median EDSS score at the time of LP was 1.5 (1 in bMS vs. 3.5; aMS, p=0.002). The median EDSS at the last follow-up was 1.5 (IQR 1.25–1.5) for the bMS group and 7.5 (IQR 7–8) for the aMS group. Twelve patients in the aMS group developed Secondary Progressive MS (SPMS), whereas none in the bMS group developed this clinical form. With regard to corticosteroid treatment before the LP, 6 out of the 35 received corticosteroids before the LP, with the last dose being within the last 3 months in 3 cases (34, 76, and 79 days before LP, respectively). In the remaining cases, the time interval exceeded 6 months. The patient who received corticosteroids 34 days before is part of the bMS group. None of the patients were on DMTs at the time of the LP; however, one patient in the aMS group was receiving azathioprine treatment.

The clinical characteristics and comparison tests between the groups are summarized in **Table 1**.

**Table 1:** Clinical characteristics and group comparisons.

	All patients	Benign MS	Aggressive MS	p value
**Number of patients**	35	20	15	
**Age at MS onset, years (mean ± SD)**	31.5 ± 9.45	28.33 ± 4.29	35.74 ± 12.58	0.15
**Female, n (%)**	21 (60%)	14 (70%)	7 (46.7%)	0.16
**Total follow-up time, years [median (IQR)]**	19.79 (15.89–24.57)	17.33 (14.54–22.18)	21.6 (18.55–24.77)	0.06
**ARR [median (IQR)]**	0.28 (0.16-0.40)	0.23 (0.15-0.30)	0.40 (0.16-0.56)	0.015*
**EDSS at last follow-up [median (IQR)]**	2 (1.5–7.5)	1.5 (1.25–1.5)	7.5 (7–8)	<0.001*
**MSSS at last follow-up (mean ± SD)**	3.8 ± 3.68	0.73 ± 0.41	7.89 ± 1.14	<0.001*
**ARMSS score at last follow-up (mean ± SD)**	4.71 ± 3.52	1.84 ± 0.95	8.54 ± 1.2	<0.001*
**DMT during follow-up, n (%):** **-No DMT** **-Moderate efficacy** **DMT** **-High efficacy DMT**	5 (14.3%) 19 (54.3%) 11 (31.4%)	4 (20%) 16 (80%) 0 (0%)	1 (6.7%) 3 (20%) 11 (73.3%)	<0.001*
**SPMS, n (%)**	12 (34.3%)	0 (0%)	12 (80%)	<0.001*
**Age at LP, years (mean ± SD)**	34.75 ± 9.08	31.05 ± 4.85	39.69 ± 11.06	0.018*
**EDSS at LP (median (IQR))**	1.5 (1–3.5)	1 (0.5–1.5)	3.5 (2–3.5)	<0.001*
**Time from MS onset to LP, months (median (IQR))**	29.96 (4.57–51.81)	24.21 (3.53–42.45)	39.49 (4.57-64.1)	0.3
**Time from previous relapse to LP, months [median (IQR)]**	2.99 (1.12–6.67)	2.14 (0.92–5.8)	3.48 (1.71–11.73)	0.14

MS, multiple sclerosis; SD, standard deviation; IQR, interquartile range; ARR, annualized relapse rate; EDSS, expanded disability status scale; MSSS, multiple sclerosis severity score; ARMSS score, age related multiple sclerosis severity score; DMT, disease-modifying treatment; SPMS, secondary progressive multiple sclerosis; LP, lumbar puncture.

*statistically significant.

### 3.2 Biomarkers and prognostic role of kFLC index

**Figure 1:**
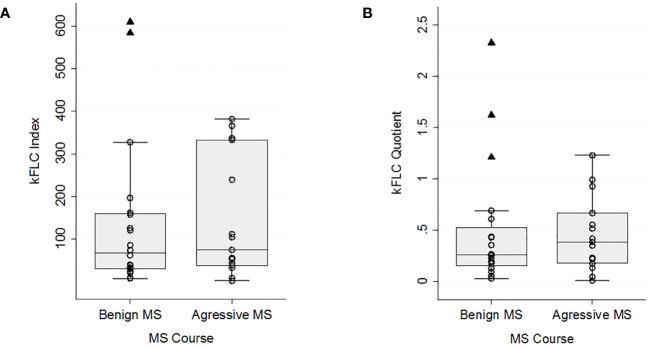
kFLC index **(A)** and kFLC quotient **(B)** distribution boxplots for benign and aggressive MS courses. A cases scatter plot is represented by dots and outliers by triangles.

The median kFLC index was slightly higher in the aMS group (74 [IQR 37.61-332.45]) than in the bMS group (67.27 [IQR 30.23-159.41]). Similarly, the kFLC quotient was also higher in the aMS group (0.39 [IQR 0.18-0.67] vs. 0.26 [IQR 0.15-0.52]). It’s worth noting that in the bMS group, there were 2 outlier values for the kFLC index and 3 for the kFLC quotient. Notably, one of these outliers, pertaining to both metrics, corresponds to the patient who had received corticotherapy 34 days prior to the lumbar puncture. The remaining outliers were not receiving any DMT, immunosuppressive treatment, or had received corticosteroids in the past 6 months. **Figure 1** shows the kFLC quotient and kFCL index distribution boxplot. The differences between groups were not statistically significant, neither in the case of the kFLC index (p=0.59) nor in the case of the kFLC quotient (p=0.64). The calculation of ROC curves showed an AUC of 0.55 (IQR 0.35-0.75) for the kFLC index and an AUC of 0.55 (IQR 0.35-0.75) for the kFLC ratio.Biomarkers and comparison tests between groups are summarized in **Table 2**.

**Table 2:** Biomarkers and groups comparison.

	All patients	Benign MS	Aggressive MS	p value
**Number of patients**	35	20	15	
**Serum kFLC, mg/L (median (IQR))**	14.8 (14.19–16.24)	14.77 (13.46–16.75)	15 (14.36–16.24)	0.69
**CSF kFLC, mg/L [median (IQR)]**	5.18 (2.49–9.21)	4.51 (2.14–9.26)	5.47 (2.86–8.12)	0.71
**kFLC quotient [median (IQR)]**	0.35 (0.18–0.61)	0.26 (0.15–0.52)	0.39 (0.18–0.67)	0.64
**kFLC index [median (IQR)]**	73.34 (32.15–196.17)	67.27 (30.23–159.41)	74 (37.61–332.45)	0.59
**kFLC index >50, n (%)**	21 (60%)	11(55%)	10(66.7%)	0.49
**kFLC index>100, n (%)**	15(42.9%)	8 (40%)	7(46.7%)	0.69
**IgG index [median (IQR)]**	0.83 (0.62–1.4)	0.72 (0.62–1.14)	0.96 (0.67–1.49)	0.2
**Positive IgG index (≥0.7), n (%)**	22 (62.9%)	11(55%)	11(73.3%)	0.312
**OCB, n (%)**	31(88.6%)	18(90%)	13(86.7%)	1

kFLC, kappa free light chains; IQR, interquartile range; CSF, cerebrospinal fluid; IgG, immunoglobulin G; OCB, oligoclonal bands.

**Figure 2:**
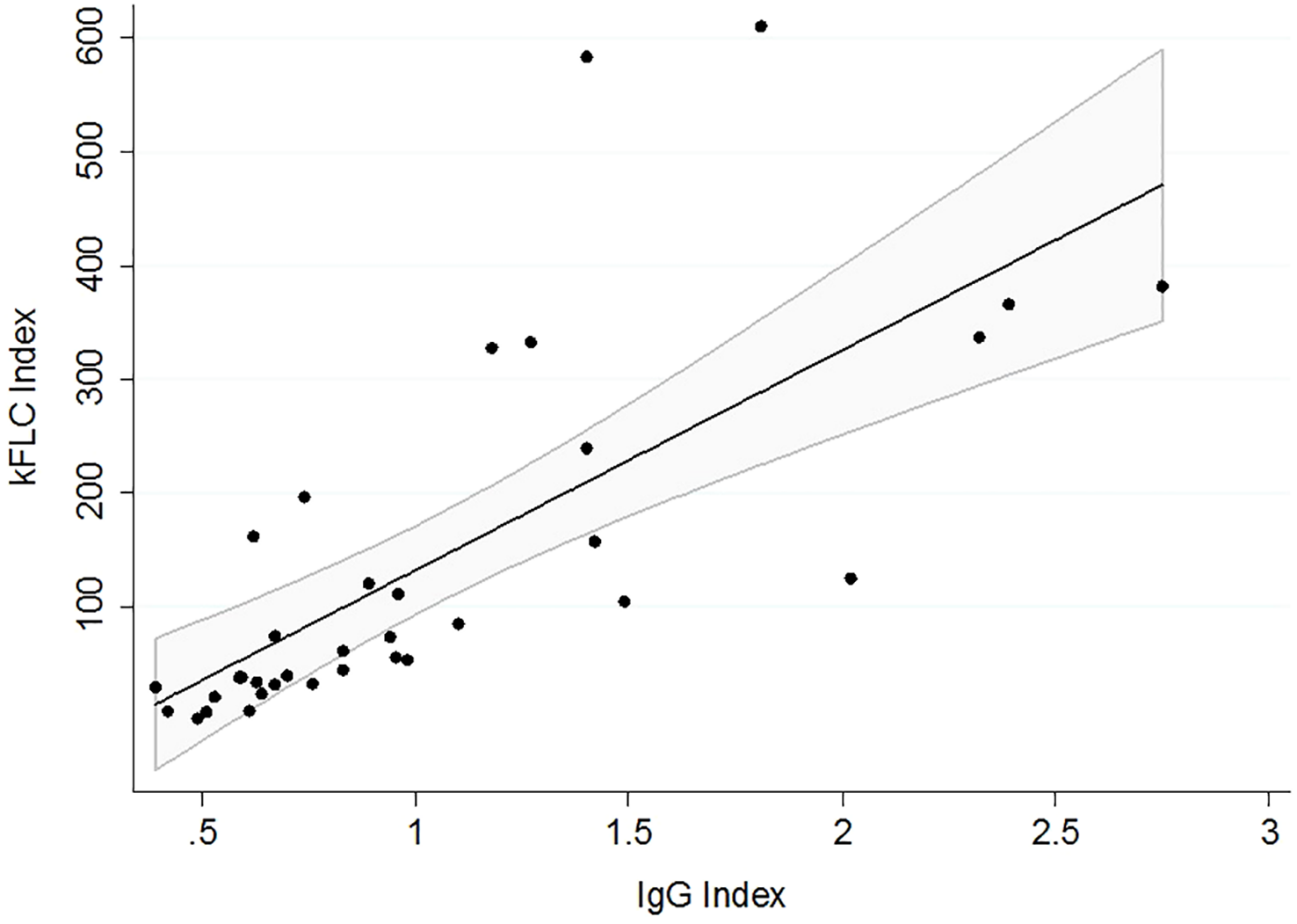
Correlation between kFLC and IgG indices. The linear regression fit is represented by a 95% confidence interval.

The IgG index was positive in 62.9% of the patients (55% for bMS vs. 73.3% for aMS, p>0.05), and the OCB was positive in 88.6% (90% for bMS vs. 86.7% for aMS, p>0.05). A strong correlation was found between the IgG and kFLC indices (r_s_ = 0.85, p<0.001), as shown in **Figure 2**.

No correlation was found between the kFLC index and the time from MS onset to LP or between the kFLC index and the time from previous relapse to LP.

The statistical analysis was repeated, excluding outliers in the bMS group, and the results were identical (data not shown).

## 4 Discussion

Recently, the kFLC index has been shown to be an alternative to OCBs for the diagnosis of MS (17, 18). It is a marker that reflects intrathecal immunoglobulin synthesis; however, unlike OCB, it is quantitative. This fact raises the question of whether, in addition to its role as a diagnostic marker, FLC could also have a role as a prognostic marker, namely, whether high FLC index values are related to a worse disease course. However, this hypothesis has not yet been fully explored.

This study aimed to determine the utility of the kFLC index in long-term prognosis. The rationale for its design was based on the fact that if the kFLC index is a useful and reliable prognostic biomarker, it should be able to differentiate patients with MS with opposite courses. No differences were observed between the two groups. Therefore, our results suggest that this index is not a useful clinical marker for long-term prognosis.

To date, some studies have assessed the relationship between the FLC index and prognosis. These can be divided into two main groups based on their objectives. The first group includes studies aimed at determining whether the FLC index is able to predict the second relapse of the disease and when it occurs; in other words, the conversion from clinically isolated syndrome to clinically definite multiple sclerosis (26–29). The second group focused on inflammatory activity in the early years (beyond the first relapse) and cumulative disability during this period.

Focusing on the second group, the oldest studies used the absolute value of KFLC in the CSF or CSF/serum FLC quotient as a metric of FLC and ELISA and radioimmunoassay techniques for its measurement (30–32).

Although they used a different metric, it is worth mentioning a study that demonstrated the relationship between the kFLC:ΛFLC ratio and EDSS at 5 years of follow-up (33).

Among the most recent studies that used the kFLC index and modern turbidimetry or nephelometry methods, three found no relationship between the kFLC index and disability progression (33–35), although one showed a shorter time to DMT initiation in patients with a higher kFLC index (35). The remaining studies found an association between a higher kFLC index and a higher confirmed EDSS (20–22), but the follow-up time in these studies was focused on the mid-term.

It is important to highlight that out of all these studies aimed at assessing the FLC prognostic capacity of disability accumulation, only two studies did not include CIS in their cohorts (22, 31), and another two analyzed the prognostic capacity of FLC in the MS subgroup, excluding CIS from the analysis (32, 34).

Due to the design of this study, a negative result does not completely rule out the possibility that differences in the kFLC index due to the disease course can be found with a larger sample size. But this design allows us to state that, even in this case, it is unlikely that this result would be relevant, and that the kFLC index would be a strong prognostic and clinically useful marker with good sensitivity, specificity, and positive and negative predictive values. As an additional *post-hoc* analysis, we tested whether the bMS and aMS groups had unequal numbers of high kFLC index values; we did so considering kFLC index cutoff values of 50 and 100, based on two recent positive studies (22, 35). There was no statistically significant difference in the number of high kFLC index values between the groups.

Moreover, we must consider that studies with a shorter follow-up time mainly assessed the accumulated disability derived from inflammatory activity, while in our study, with a median follow-up of approximately 20 years, it is more likely that the progressive phase of the disease had a greater influence on the disability of our patients. It is worth mentioning that 12 out of the 15 patients from the aMS group in our study progressed to the secondary progressive phase during follow-up. In fact, some studies have suggested that the kFLC index could be related to the CNS-resident B lymphocyte load, which gives it biological plausibility (22, 33, 36). This supports the hypothesis that the kFLC index can predict the more inflammation-dependent phase of the disease but not the progressive phase, which is more dependent on neurodegeneration pathogenically associated with slow or smoldering plaques, macrophages, and microglia (9, 37). Ultimately, this could explain the difference in results between previous studies and our long-term study.

Another distinguishing factor worth highlighting in comparison to numerous previous studies, which could explain the difference in results, is the fact that in our study, only patients diagnosed with MS according to the McDonald 2017 criteria have been included. This was done to avoid mixing the diagnostic capacity of kFLC (its ability to predict whether CIS will convert to MS) with its prognostic capacity for disability. In fact, out of the 4 studies that analyze the prognostic capacity of disability without including CIS or excluding the CIS subgroup from the prognostic analysis, only one finds a relationship with the kFLC index employing modern detection tecnhiques, but with a median follow-up time of less than 5 years (22).

Whether the proximity of a recurrence or the use of immunosuppressive drugs affects kFLC index values has been discussed, and there are a few previous studies that provide some data in this regard (27). In our cohort, owing to the selection criteria, none of the patients received corticosteroids in the month before sample collection. We were unable to demonstrate a relationship between the time from the previous relapse to sample collection and the kFLC index.

With regard to the limitations of this study, this was conceived as an exploratory observational study. This could lead to a bias associated with observational studies. The sample size represents another limitation of the study as it can result in both a lack of statistical power and potential biases.

Nevertheless, as we have mentioned previously, we believe that due to the study’s design, its outcome is still valuable as it reasonably allows us to rule out the actual clinical utility of FLC as a long-term prognostic marker. Although no significant differences were observed in the age at onset between the groups, a significant difference was noted between the ages of the patients at the time of LP, which was higher in the aMS group than in the bMS group. According to FLC kinetics, older age should contribute to an increase in FLC serum levels, as multiple comorbidities as well as renal function impairment can raise them (38–40), although it is unclear if this would influence the kFLC index. In any case, this would favor finding higher levels of the kFLC index in the aMS group and lower levels in the bMS group, that is, it would facilitate finding differences between groups. Given that no such differences were observed in our study, it is unlikely that this played a role. MRI data were not included because they were not uniformly available across the sample, which constitutes another limitation of this study.

In conclusion, the levels of kFLC did not differ between patients with aggressive and benign MS in the long term. Our approach can be used as an efficient way to select candidate biomarkers for the long-term prognosis of MS.

## Data availability statement

The raw data supporting the conclusions of this article will be made available by the authors, without undue reservation.

## Ethics statement

The studies involving humans were approved by Ethics Committee of the Hospital Universitari de Bellvitge (reference PR134/22). The studies were conducted in accordance with the local legislation and institutional requirements. The participants provided their written informed consent to participate in this study.

## Author contributions

PA-P contributed to the collection, analysis, and interpretation of the data and writing of the report. LG-S, FM, BU, VM, and MF contributed to the analysis and interpretation of the data, and correction of the report. IL contributed to data collection. AM-V, EM, and LR-P contributed to the data collection and report correction. AM-Y and SM-Y contributed to the study design, data interpretation, and report correction. LB contributed to the study design; collection, analysis, and interpretation of data; and correction of the report. All authors contributed to the article and approved the submitted version. 
